# Synthesis of Au/CdSe Janus Nanoparticles with Efficient Charge Transfer for Improving Photocatalytic Hydrogen Generation

**DOI:** 10.1186/s11671-019-3185-6

**Published:** 2019-11-27

**Authors:** Xiao-Dan Liu, Kai Chen, Song Ma, Zhong-Hua Hao, Shan Liang, Li Zhou, Qu-Quan Wang

**Affiliations:** 1 Key Laboratory of Artificial Micro- and Nano-structures of the Ministry of Education, School of Physics and Technology, Wuhan University, Wuhan, 430072 People’s Republic of China; 2 The Institute for Advanced Studies, Wuhan University, Wuhan, 430072 People’s Republic of China; 3 Department of Physics, Hunan Normal University, Changsha, 410081 People’s Republic of China

**Keywords:** Au/CdSe Janus nanospheres, Interface, Highly efficient photocatalysis

## Abstract

Metal-semiconductor heterostructures integrate multiply functionalities beyond those of their individual counterparts. Great efforts have been devoted to synthesize heterostructures with controlled morphologies for the applications ranging from photocatalysis to photonic nanodevices. Beyond the morphologies, the interface between two counterparts also significantly influences the performance of the heterostructures. Here, we synthesize Au/CdSe Janus nanostructures consisting of two half spheres of Au and CdSe separated by a flat and high-quality interface. Au/CdSe with other morphologies could also be prepared by adjusting the overgrowth conditions. The photocatalytic hydrogen generation of the Au/CdSe Janus nanospheres is measured to be 3.9 times higher than that of the controlled samples with CdSe half-shells overgrown on the Au nanospheres. The highly efficient charge transfer across the interface between Au and CdSe contributes to the improved photocatalytic performance. Our studies may find the applications in the design of heterostructures with highly efficient photocatalytic activity.

## Introduction

Metal-semiconductor colloidal heterostructures have attracted extensive interests due to their extraordinary optical behaviors and functionalities far beyond those of their individual counterparts and have exhibited great potential in solar energy conversion [1, 2], photocatalysis [3–8], photoelectric devices [9–11], and photothermal therapy [12–15], etc. Especially, plasmon-based hybrid nanostructures become a promising candidate for photocatalytic water splitting or hydrogen generation with excellent photocatalytic performance [16–19]. Colloidal nanoparticles of metal chalcogenide semiconductors (sulfide, selenide, and telluride) have received significant attention in photocatalytic application due to their suitable and tunable band gap matched with solar spectrum as well as their chemical properties. However, the low absorption efficiency in visible light region and the quick recombination of photo-induced charge carriers have limit the application of pure semiconductor nanoparticles. To overcome these issues, many efforts have been devoted to integrate plasmonic metal nanocrystals (nanospheres [20], nanorods [21], nanoplates [22], etc.) and chalcogenide semiconductors (CdX [23–28], Ag_2_X [29–33], Cu_2_X [12–15], PbX [34] etc. (X = S, Se, Te)) to build hybrid nanostructures with intriguing properties.

As for the plasmon-enhanced photocatalytic performance, many possible mechanisms have been discussed in previous works, including effectively harvesting light energy through surface plasmon resonances, concentrating local electromagnetic field in adjacent semiconductors, promoting photoexcited charge generation and transfer, suppressing electron-hole recombination and plasmon-induced hot-electron transfer from metals to semiconductors [35–39]. Besides that, several structural factors such as morphology, size, hybrid configuration, and contact interface have been reported to be crucial for photocatalytic activity [40–43]. Zhao et al. have finely tuned the structural symmetry of the Au/CdX (X = S, Se, Te) hybrid nanoparticles with controllable spatial distribution between the two components by a non-epitaxial synthetic route and demonstrated the dependence of photocatalysis on the structural symmetry [41]. The interfacial charge transfer and the exposure of active materials to reaction solution are the important factors for determining the performance of heterodimer type and core-shell-type hybrids [41, 44]. The possibility of charge transfer between the metal and the chalcogenide semiconductors has been exhibited in several types of hybrids [41, 44–46]. Additionally, the charge transfer also depends significantly on the interfacial conditions, such as interfacial energy and quality between the two counterparts [41, 44]. There remain great challenges to obtain a good heterogeneous interface for metal-semiconductor hybrid nanostructures due to the large lattice mismatch between two components. Therefore, it is meaningful to finely tailor the interface and contact to achieve the tunable properties and electronic mobility in the metal-semiconductor hybrid nanostructures.

In this paper, we report a particular approach to synthesize water-dispersed asymmetric Au/CdSe Janus heterostructures with a flat and high-quality interface between Au and CdSe. By manipulating the pH value of the reaction solution, CdSe with different morphologies and coverages are grown on the Au nanoparticles. The results show the pH value is crucial for the formation of Janus morphology with the flat and high-quality interface. Hydrogen generation measurements show that the Janus Au/CdSe heterostructures has a significantly higher efficiency than those of the other types of hybrid structures due to the low interface energy and the improved electron transfer efficiency on the interface of Au and CdSe.

## Methods/Experimental

### Materials

Chloroauric acid (HAuCl_4_·4H_2_O, 99.99%), silver nitrate (AgNO_3_, 99.8%), glycine acid (99.5%), selenium powder (Se, 99.5%), L-ascorbic acid (99.7%), sodium hydrate (NaOH, 96.0%), cadmium nitrate tetrahydrate (Cd(NO_3_)_2_·4H_2_O, 99.0%), hydrochloric acid (HCl, 36–38%), hexamethylenetetramine (HMT, 99.0%), and sodium borohydride (NaBH_4_, 96%) were all purchased from Sinopharm Chemical Reagent Co. Ltd. (Shanghai, China). Cetyltrimethylammonium-bromide (CTAB, 99.0%) was obtained from Amresco, Inc. (America). All chemicals were used as received and without further purification.

### Synthesis of Au Nanoparticles

The CTAB-stabilized Au nanoparticles were synthesized at room temperature by a seed-mediated growth method reported previously [20]. Firstly, 4.5 mL aqueous solution was prepared by mixing 500 μL of 5 mM HAuCl_4_ and 5 mL of 0.2 mM CTAB, and then 600 μL of 10 mM ice-cooled NaBH_4_ solution was added. The brownish solution of Au seeds was left undisturbed for 2 h for further use. Next, 120 μL Au seed solution was added into a aqueous mixture including 190 mL of H_2_O, 4 mL of 10 mM HAuCl_4_, 9.75 mL of 0.1 M CTAB, and 15 mL of 100 mM ascorbic acid. The solution was well mixed by a slight shaking and then was allowed to stand overnight for the growth of Au nanoparticles.

### Synthesis of Au-Ag Bimetallic Nanoparticles

Firstly, the pH value of a aqueous mixture including 5.0 mL of the Au nanoparticles (8.0 nM) and 5.0 mL of 200 mM glycine acid was respectively adjusted to 2.5, 4.5, 7.2 or 8.1 by the dropwise addition of HCl solution (V_HCl_:V_H2O_ = 1:9) or NaOH solution (2 M). The mixture was kept at 30 °C under stirring for 1 min. Then, 15 µL of 100 mM AgNO_3_ solution was injected. The mixture was kept at 30 °C without stirring for 10 h. The products of Au-Ag bimetallic nanoparticles were directly used for the growth of Au-CdSe hybrid nanoparticles.

### Synthesis of Au/CdSe Janus Heterostructures

The Au/CdSe Janus heterostructures was prepared by mixing 2 mL of the as-prepared Au-Ag nanoparticles, 6 mg of selenium powder, 0.01 mL of 100 mM Cd(NO_3_)_2_ solution, and 40 µL of 10 mM NaBH_4_ solution. The mixed reaction was vigorously stirred at 90 °C for 2 h. The products were centrifuged at 9500 rpm for 5 min and washed by water twice. The controlled samples with other morphologies were prepared by the same procedure except for the pH value of the growth of Au-Ag nanoparticles.

### Evaluation of Photocatalytic Activities

The visible light photocatalytic hydrogen evolution tests were carried out in a quartz tube reactor with a rubber diaphragm. One hundred milligrams of Au/CdSe photocatalyst powder was dispersed in 50 mL of an aqueous solution containing 5 mL of lactic acid as a sacrificial agent in a quartz tube reactor. The reactor was pumped off with stirring for 30 min to remove any dissolved air. The light source is a 300-W xenon lamp with an ultraviolet cutoff filter (*λ* > 420 nm). During the entire photocatalytic test, the temperature of the suspension was maintained at 6 °C with an external water-cooling system to withstand the temperature rise of the optical radiation. The content of hydrogen was automatically analyzed by on-line gas chromatography (Tianmei GC-7806).

### Characterization

TEM studies were done with a JEOL 2010 HT microscope operated at 200 kV by drop casting the sample dispersions on carbon-coated copper grids. HRTEM, TEM, and EDX analyses were performed using a JEOL 2010 FET microscope operated at 200 kV accelerating voltage. The UV-Vis spectra were recorded with a TU-1810 (Purkinje General Instrument Co. Ltd. Beijing, China) and Cary 5000 (Agilent) spectrometer. All optical measurements were performed at room temperature under ambient conditions.

## Results and Discussion

**Fig. 1:**
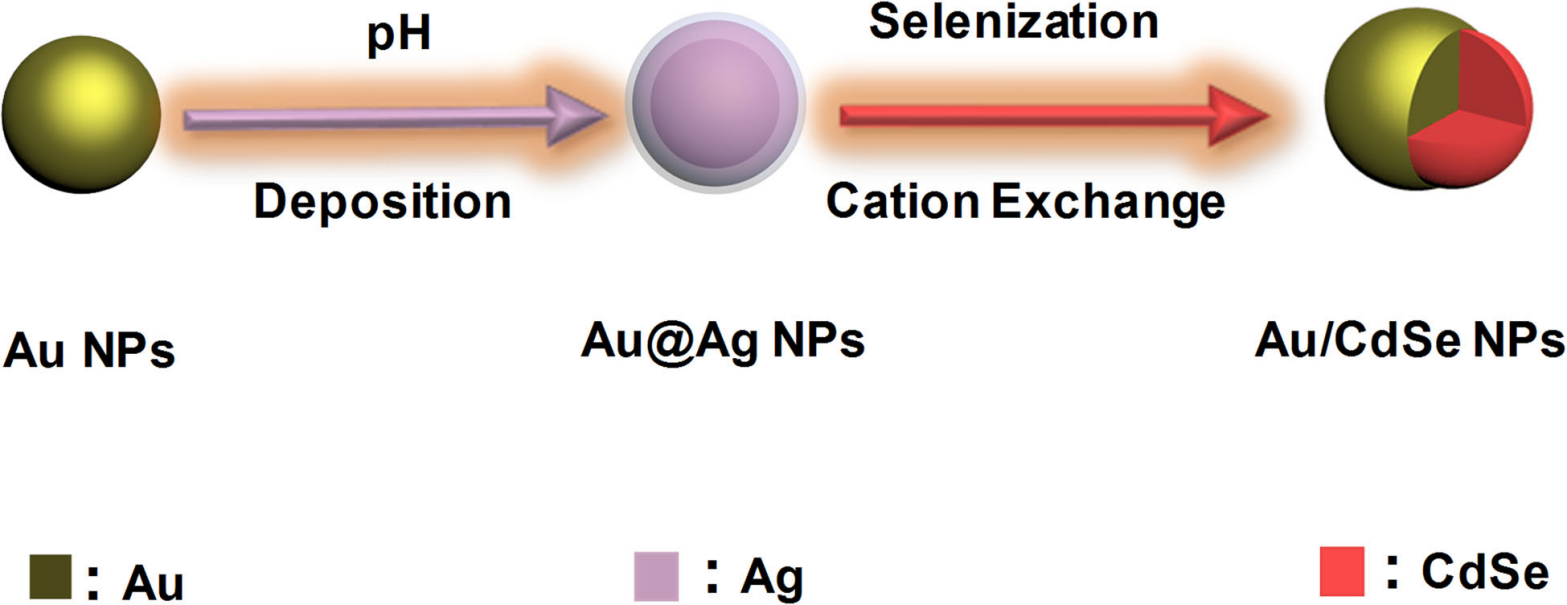
Schematic illustration for the synthesis of Au/CdSe Janus nanospheres

Figure 1 schematically describes the synthesis of water-dispersed Au/CdSe Janus nanospheres. Firstly, CTAB-stabilized Au nanoparticles were prepared by a seed-mediated growth method [20]. Then a small amount of Ag was deposited on the Au nanoparticles with controlled pH value of the reaction solution, and finally the uncentrifuged solution of Au-Ag nanoparticles was put into a reaction including selenization, cation exchange with Cd^2+^, and overgrowth of CdSe.

**Fig. 2:**
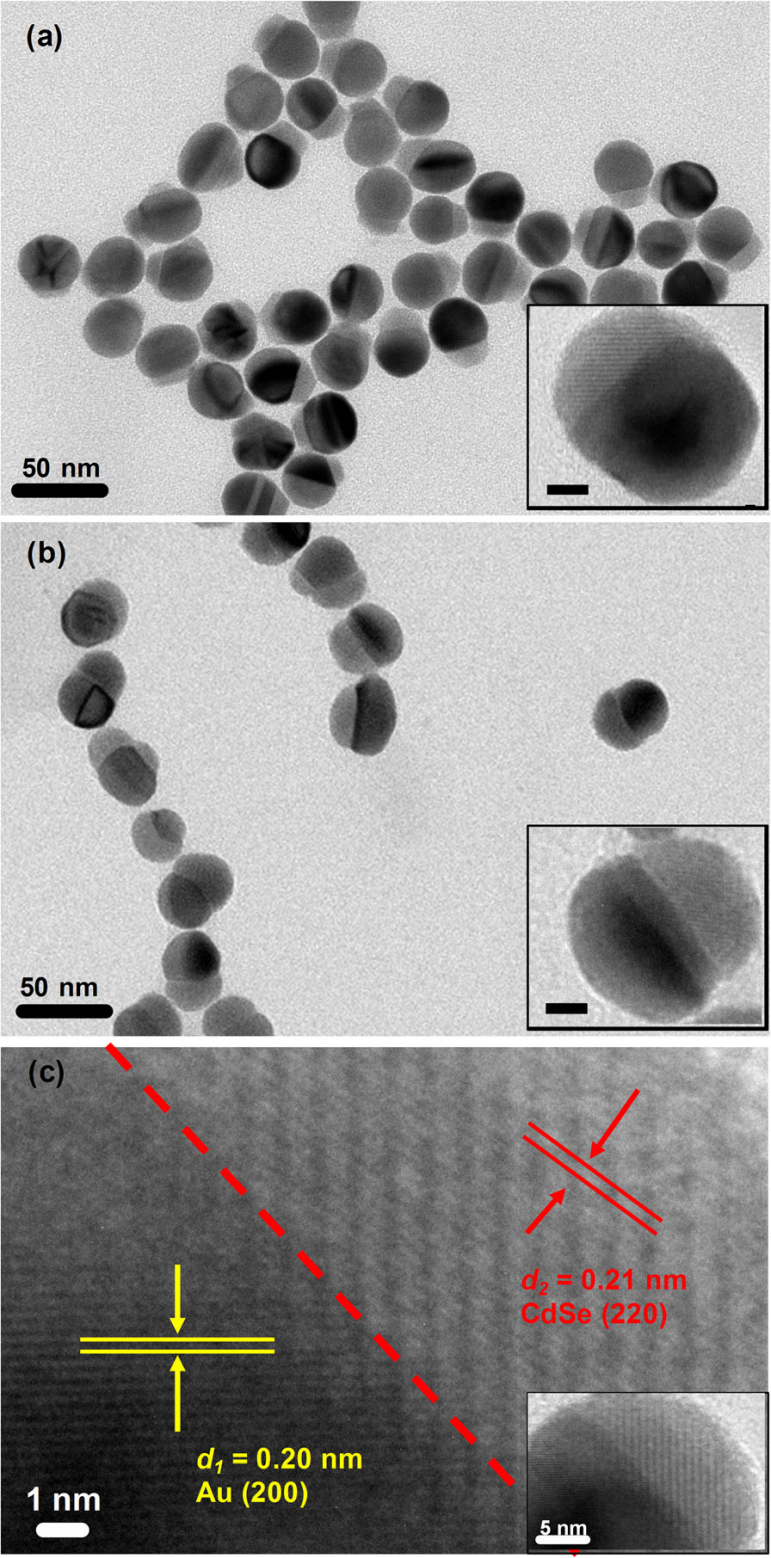
TEM images of Au/CdSe Janus nanospheres at pH = 2.5 with different reaction times. **a** 2 h. **b** 3 h. The insets show a single Janus nanosphere. The scale bars in the insets are 5 nm. **c** HRTEM image of the interfacial area of Au/CdSe Janus nanospheres

**Fig. 3:**
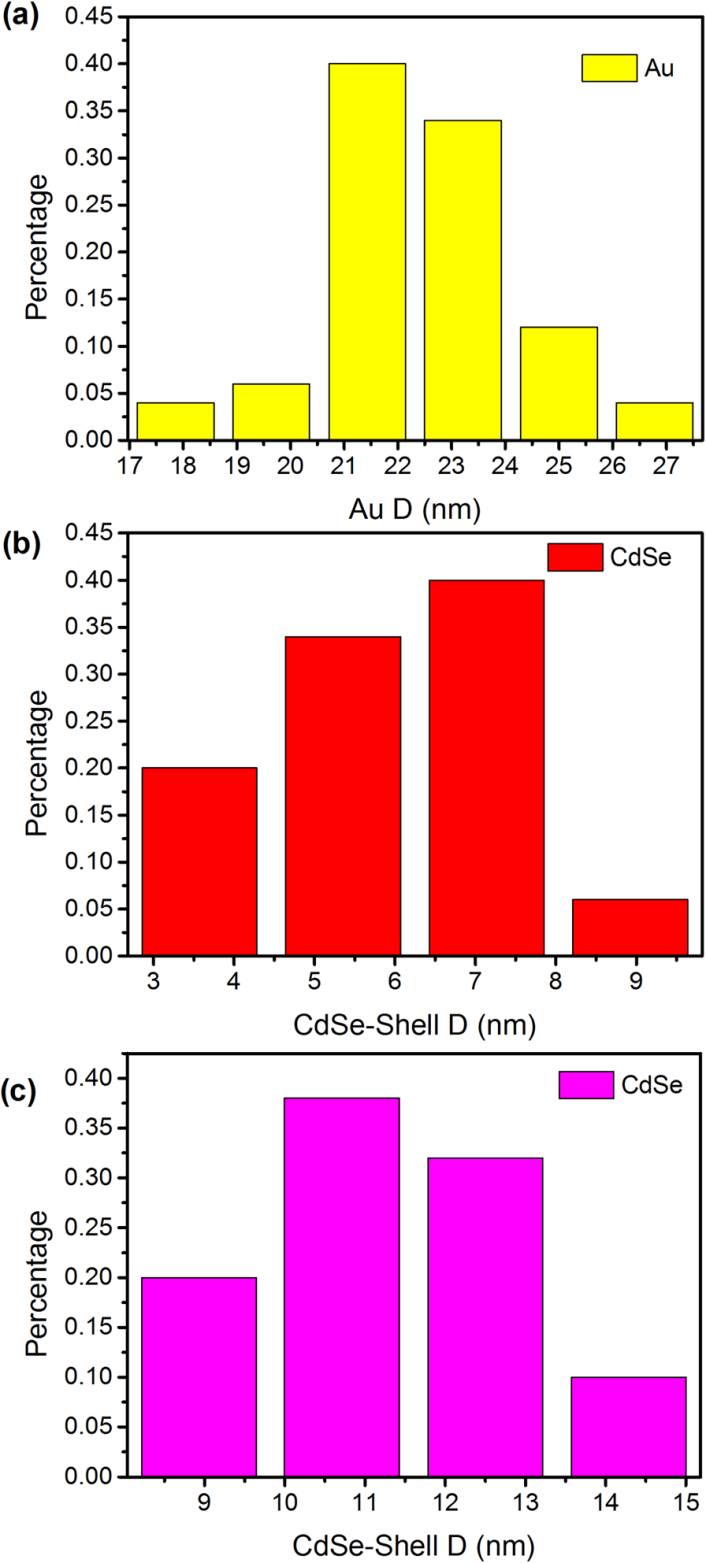
Size distribution of **a** Au nanoparticles and CdSe diameter in Au/CdSe Janus nanospheres with different reaction times. **b** 2 h. **c** 3 h. The Au/CdSe Janus nanospheres is prepared at pH = 2.5 with 0.05 mL Cd(NO_3_)_2_ (0.1 M)

**Fig. 4:**
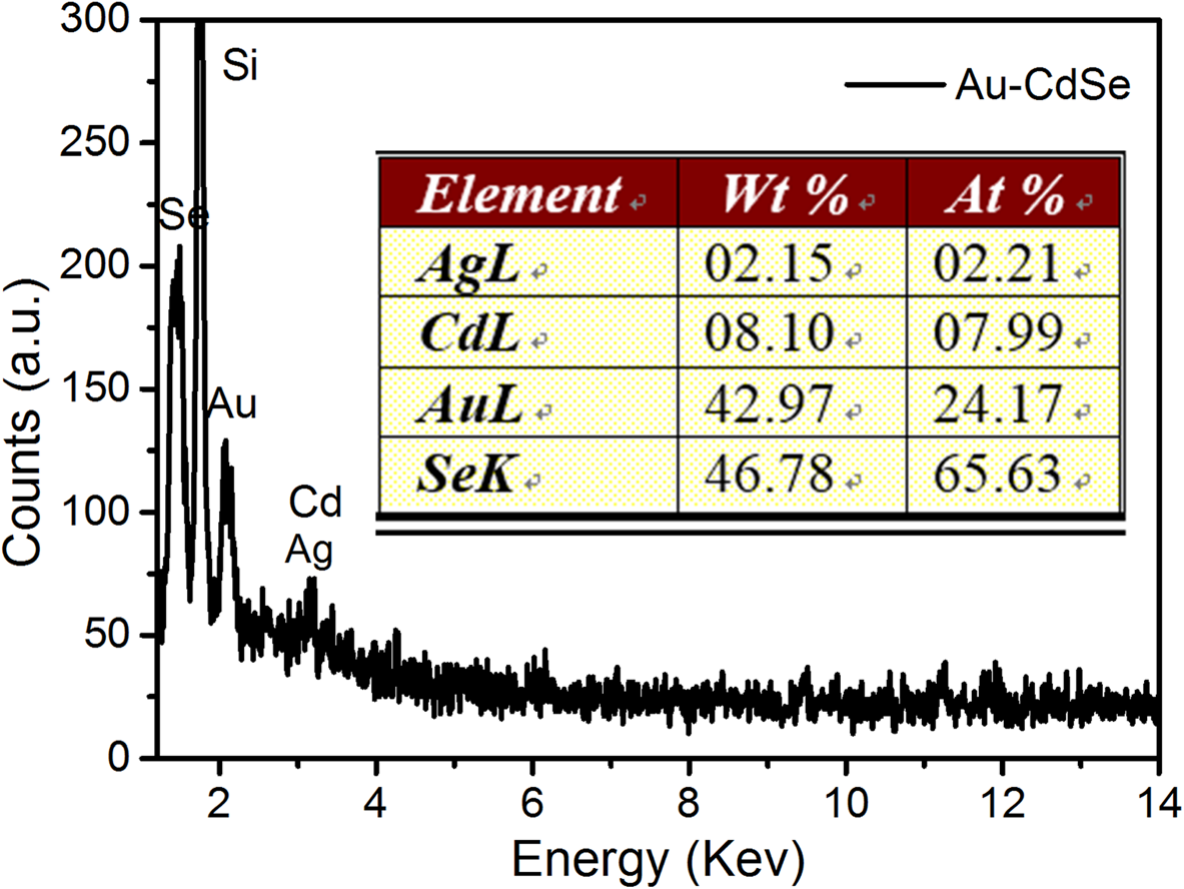
EDX spectrum of Au/CdSe Janus nanospheres dropped on a silicon wafer. The inset table is the percentage of each element

The growth processes of Au/CdSe Janus nanospheres are very similar to that of the mike-like Au-AgCdSe nanorods we previous reported [26]. In a typical process, the synthesis of Au/CdSe Janus nanospheres can be divided into three steps: Ag wetting layers deposition, Ag selenization, and CdSe selective growth. In the first step, Au-Ag bimetallic spherical nanoparticles were synthesized by consecutively adding glycine, HCl, and AgNO_3_ to an aqueous dispersion of CTAB-stabilized Au nanoparticles at 30 °C. Ag was deposited onto the CTAB-stabilized Au nanospheres by reducing AgNO_3_ with glycine acid at the pH value of 2.5 adjusted by adding appropriate HCl. The corresponding thicknesses of Ag layer can be tailored by adjusting of glycine reduction capacity with pH value. In addition, the Ag deposition possibly produces an AuAg alloyed layer rather than a pure Ag layer on the surface of Au nanoparticles due to the atom diffusion [47]. The produced Au-Ag bimetallic nanoparticles are supposed to be very important for the formation of Au/CdSe Janus nanospheres with a flat interface. Next is the selenization of Ag layers. This step is performed by sequentially adding Se powder, Cd(NO_3_)_2_, and NaBH_4_ into the uncentrifuged solution of Au-Ag nanospheres at 90 °C with stirring for 2 h. The Ag layer could be selenized spontaneously by Se powder. As the atom diffusion leads to the forming of AuAg alloyed layer coating on the Au nanoparticles, partial Au could also be selenized. This process would lead to an etching effect of Au. Once formed, Ag_2_Se will be acted as the “anchor point” for overgrowth of CdSe. The last step is the formation of Au/CdSe Janus nanospheres. Ag_2_Se ripening, cation exchange with Cd^2+^, and epitaxial growth of CdSe are supposed to be involved in the formation of Au/CdSe Janus nanospheres. Here, it should be noted that the solution remains acidic with pH = 2.5. The relative high concentration of Se^0^ and the low concentration of Se^2-^, because of the inhibited reducibility of the reducing agent in this condition, would induce a relatively fast ripening process of Ag_2_Se and slow overgrowth of CdSe. Meanwhile, the conductive metal nanosphere can further offer an effective pathway for electron transfer in Ag_2_Se ripening process, which will eventually lead to a hemispherical nanoshell. The subsequent cation exchange with Cd^2+^ ions produces a CdSe layer, which facilitates the overgrowth of CdSe on these sites, overcoming the barrier of crystal lattice mismatch. The obtained Au/CdSe Janus nanospheres composed of two hemispheres are clearly observed in Fig. 2a. In addition, since Ag layers are very thin at pH = 2.5, it can be imagined that the selenization of the Ag layer and the ripening of Ag_2_Se are a short process. Then, it is inevitable that Se^0^ will continue to etch the AuAg alloyed interface. The metal-semiconductor interfaces would further flatten along a certain crystal plane [48]. Meanwhile, the corresponding semiconductor bumps gradually enlarge, as displayed in Fig. 2b. The initial Au nanoparticles have an average diameter of 22 ± 2 nm, as displayed in Fig. 3a. After the coating of CdSe nanocrystals with stirring for 2 h at 90 °C, the thickness of the semiconductor hemispheres is 6 ± 2 nm (Fig. 3b). As the reaction continues for another hour, the size of the semiconductor counterpart is increased by 5 ± 1 nm (Fig. 3c), implying the further large overgrowth of CdSe. Figure 2c shows the HRTEM image of a single Au/CdSe Janus nanosphere. The lattice plane spacings of 0.20 nm and 0.21 nm agree well with the (200) lattice planes of the fcc gold crystal [49] and the (220) planes of CdSe [26]. The EDX spectrum in Fig. 4 also indicates the composition of Au, Cd, and Se in the Janus nanospheres as well as the residual Ag species.

**Fig. 5:**
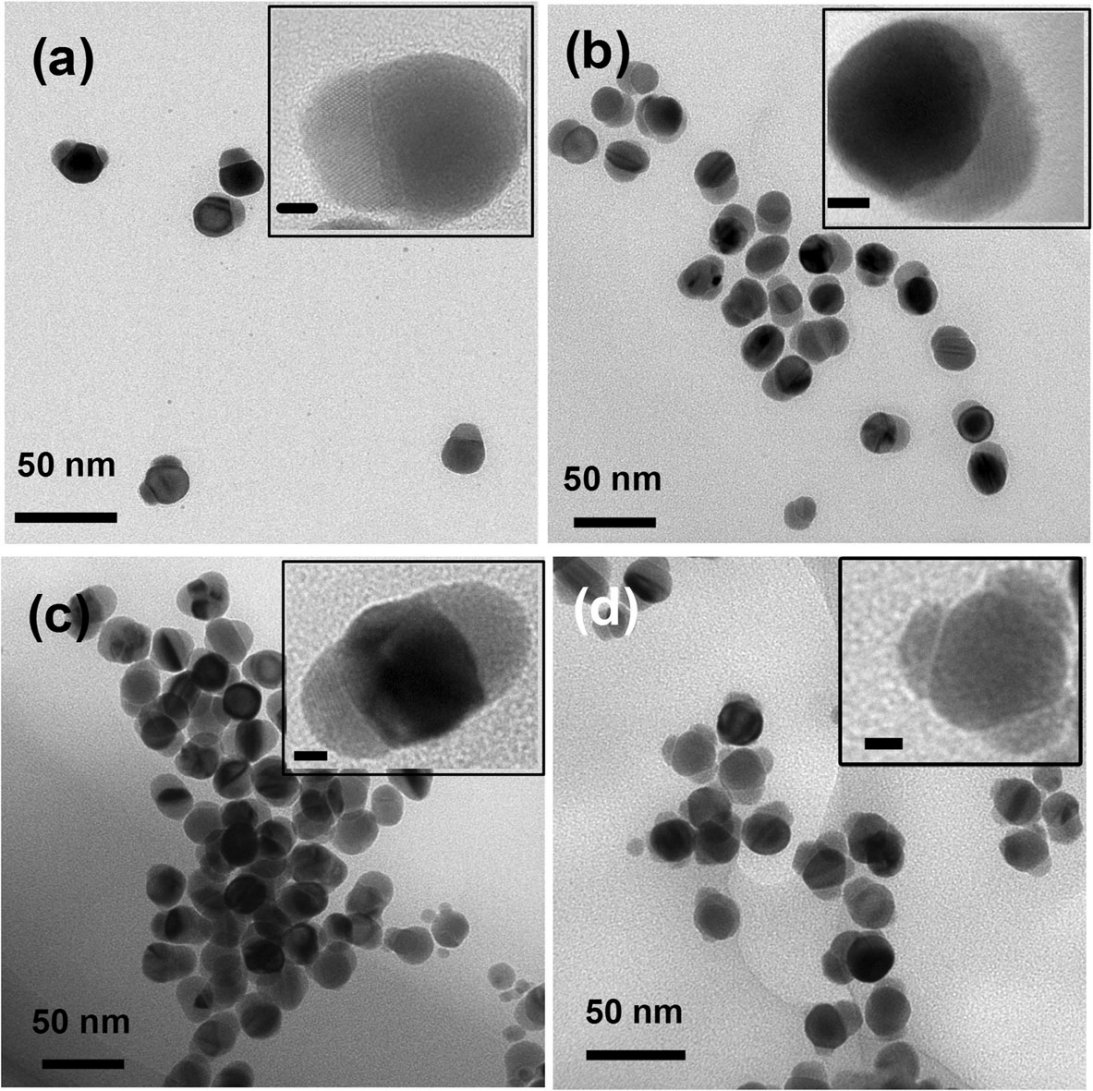
TEM images of four different types of Au/CdSe hybrid nanoparticles. **a** Janus nanospheres. **b** Heterodimers. **c** Symmetric double-headed nanoparticles. **d** Multi-headed nanoparticles. The hybrids are synthesized through manipulating the pH value of Ag deposition (2.5, 4.5, 7.2, 8.1, respectively) with the same amount of Cd(NO_3_)_2_ (0.05 mL and 0.1 M). The scale bars in the insets are 5 nm

Due to the lattice mismatch, the hetero interface is strongly influenced by the adhesion of the capping ligand, surfactant, precursor, and solvent in the colloidal phase [50–52]. Several issues should be considered to understand the morphology evolution of Au/CdSe hybrid nanoparticles. In the preparation process of Au/CdSe hybrid nanoparticles, the pH value in the first step is a key factor for well controlling the reaction kinetics. When the pH is increased, the reducing power of BH^4−^ is boost up. It will induce the increase of Se^2+^ ions in the solution and promote the rapid formation of CdSe. As such, it is reasonable to assume that once the formation rate of CdSe exceeds the ripening rate of Ag_2_Se, more options will be provided for the selective growth of CdSe. In addition, since a higher pH value also makes glycine a stronger reductant in the first step, the reduction of Ag could be boosted up, and the thickness of Ag wetting layers would increase with pH value of the reaction solution. As a result, more Se atoms are allocated to the process of Ag layer selenization and CdSe growth at high pH environment, which will prolong the Ag_2_Se ripening time and ease the AuAg interfacial etching [48]. Our experiment conducted at different pH environments also confirmed this argument. As shown in Fig. 5, through manipulating the pH value (2.5, 4.5, 7.2, and 8.1, respectively) of the solution while keeping the amount of Cd(NO_3_)_2_ constant (0.05 mL and 0.1 M), four different types of Au/CdSe hybrid nanoparticles could be produced, such as Janus nanospheres, heterodimers (consisting of CdSe half-shells overgrown on the Au nanospheres), symmetric double-headed nanoparticles, and multi-headed nanoparticles. The four hybrid nanoparticles show different interfaces between Au and CdSe. Furthermore, as shown in Additional file 1: Figure S1, at low pH value, the slow growth rate of CdSe may also induce the high-degree crystallization and the more obvious anisotropic growth of the semiconductor, which could result in the low interfacial strain energy and grain boundary energy [41, 44].

**Fig. 6:**
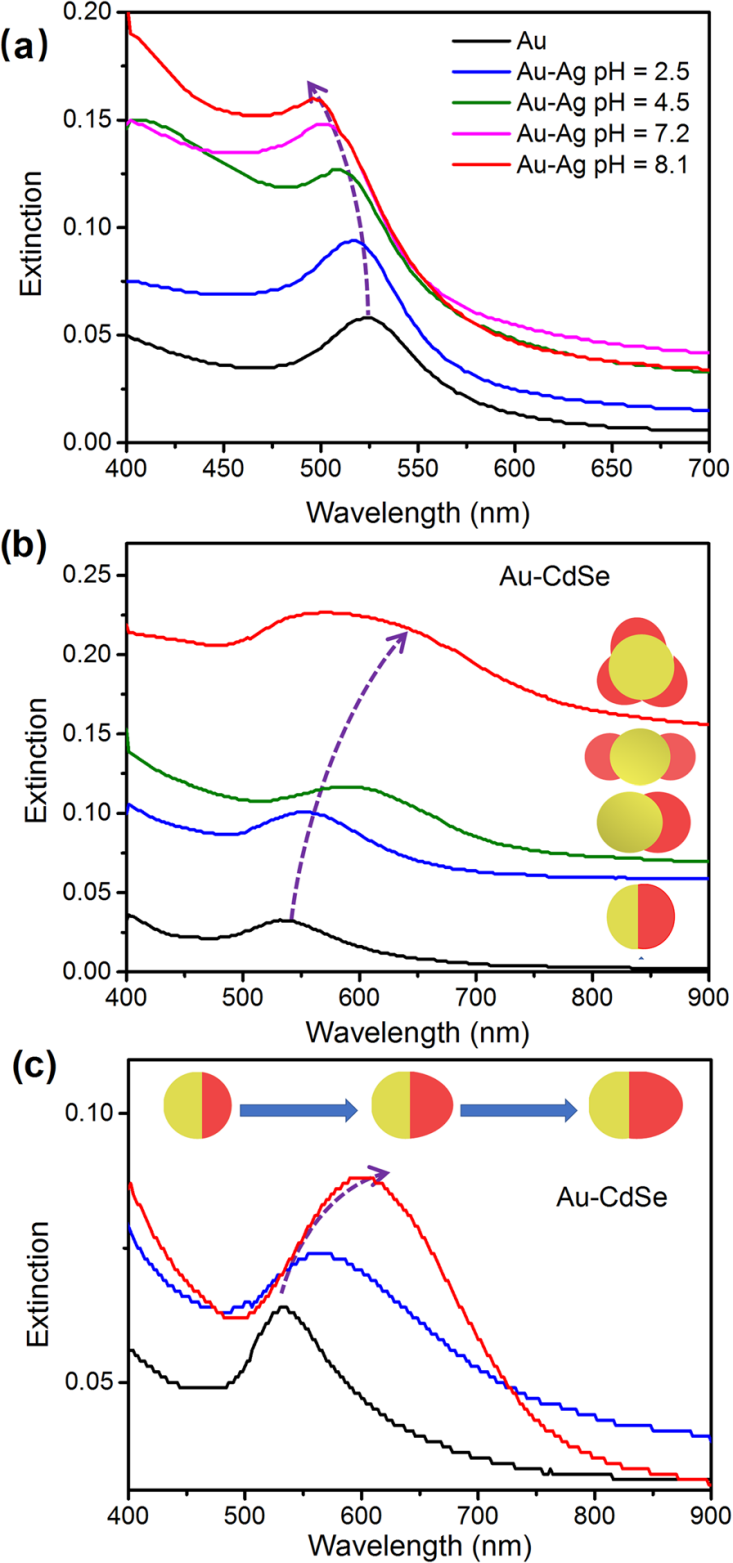
UV-vis-NIR extinction spectra of **a** Au and Au-Ag nanoparticles, **b** Au/CdSe hybrid nanoparticles with different morphologies such as Janus nanospheres (pH = 2.5), heterodimers (pH = 4.5), symmetric double-headed nanoparticles (pH = 7.2), multi-headed nanoparticles (pH = 8.1), and **c** Au/CdSe Janus nanospheres obtained with different amounts of 0.1 M Cd(NO_3_)_2_: 0.05 mL, 0.1 mL, and 0.15 mL

The Au nanoparticles display a strong SPR band located at about 522 nm. As shown in Fig. 6a, the Ag deposition leads to a blue-shit of plasmon band. As the pH value for Ag deposition is respectively set to 2.5, 4.5, 7.2, and 8.1, the absorption peak of Au-Ag blueshifts respectively to 516 nm, 508 nm, 503 nm, and 500 nm. The high growth rate of Ag at high pH value leads to the thick Ag shell and the large blueshift of plasmon band [53, 54]. Figure 6b shows the extinction spectra of the four types of Au/CdSe hybrid nanoparticles. The growth of CdSe leads to the redshift of plasmon band. As the pH value of Ag deposition is increased, the extinction band is redshifted to 536 nm, 553 nm, 594 nm, and 602 nm, respectively. The large redshift at high pH value is caused by the increased thickness and coverage of CdSe on the Au nanoparticles, and thus the increased effective refractive index environment [32, 45]. The amount of Cd(NO_3_)_2_ also influences the dimension of grown CdSe and the plasmon shift. Figure 6c shows that, at the condition of pH value of 2.5, the extinction peak of Au/CdSe Janus nanospheres is gradually redshifted from 536 to 566 nm and 605 nm as the amount of 0.1 M Cd(NO_3_)_2_ is increased from 0.05 to 0.1 mL and 0.15 mL. In addition, both in Fig. 6b and c, the extinction bands of Au/CdSe are broadened compared with the SPR characteristics of pure Au nanoparticles, which is possibly caused by the inhomogeneous distribution of CdSe thickness and coverage. Furthermore, the band gap absorption of CdSe at around 1.74 eV may arise as the CdSe is grown thicker. The presence of plasmon-exciton coupling may also contribute to the spectra broadening [41].

**Fig. 7:**
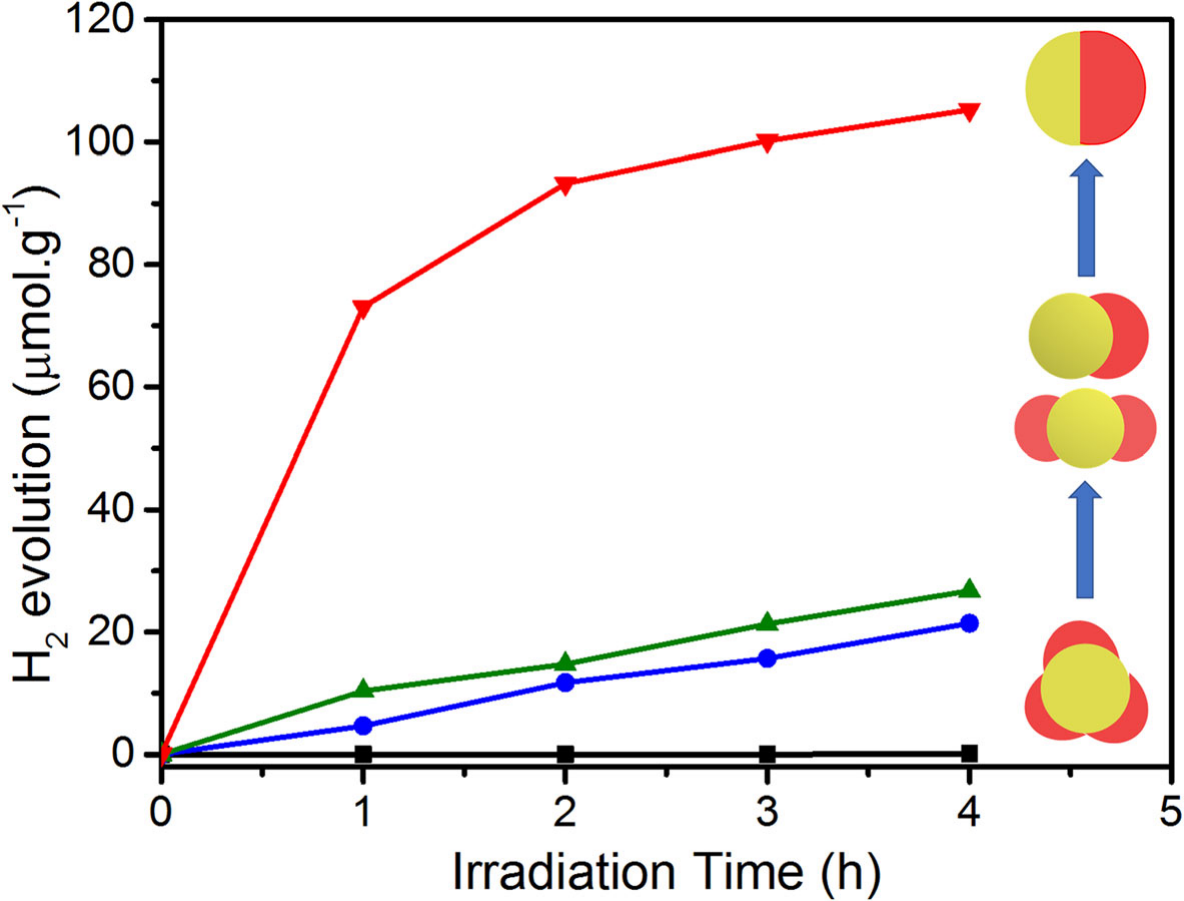
Photocatalytic activity of four different types of Au/CdSe hybrid nanoparticles such as Janus nanospheres, heterodimers, symmetric double-headed nanoparticles, multi-headed nanoparticles for H_2_ production reactions

The photocatalytic H_2_ generation of the four types of Au/CdSe hybrid nanoparticles are evaluated under a visible light illumination (*λ* > 420 nm) in 50 mL aqueous solution with 5 mL lactic acid as an environmentally friendly sacrificial agent. As shown in Fig. 7, the multi-headed nanoparticles, symmetric double-headed nanoparticles, heterodimers, and Janus nanospheres exhibit a gradually increased photocatalytic activity. The multi-headed Au/CdSe nanoparticles show a very low hydrogen production rate of 0.16 μmol h^−1^ g^−1^. Symmetric double-headed nanoparticles and heterodimers show the hydrogen production rates of 21.4 μmol h^−1^ g^−1^ and 26.7 μmol h^−1^ g^−1^, respectively. More notably, the hydrogen production rate of Au/CdSe Janus nanospheres is 105.2 μmol h^−1^ g^−1^, which is 3.94 times that of the heterodimer structures.

**Fig. 8:**
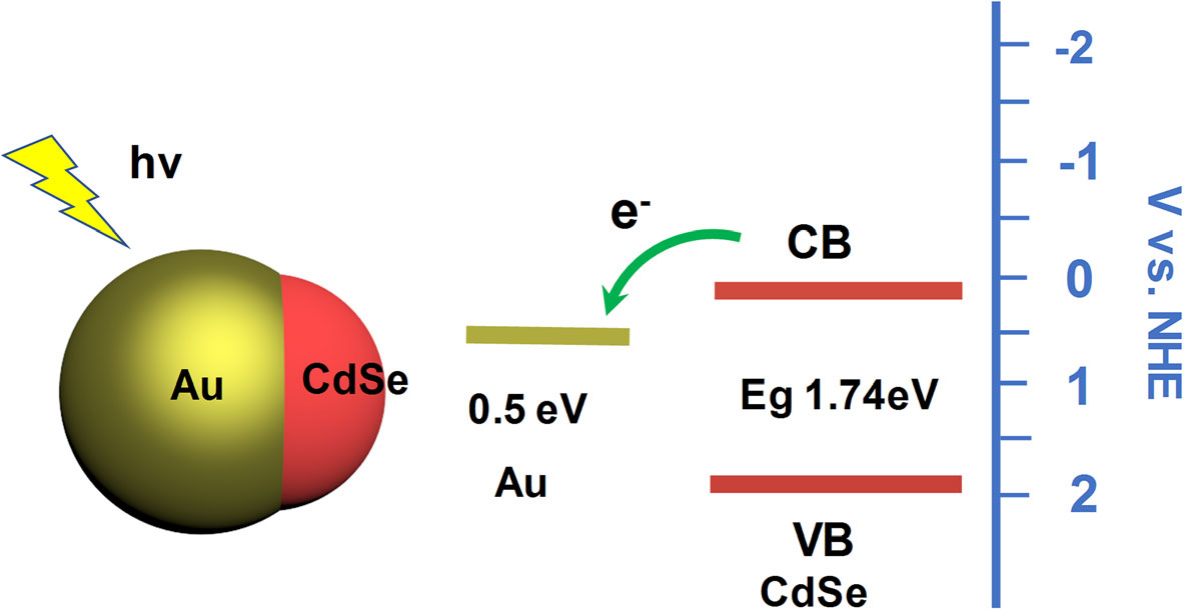
Schematic illustration of the charge separation at the interface of Au/CdSe hetero-nanostructure

The inner charge separation at the interface of the Au/CdSe heterostructure and the charge transfer processes in photocatalytic H_2_ generation are further discussed and shown in Fig. 8 to understand the mechanism of this enhanced photocatalytic activity. CdSe is a bandgap (*E*g = 1.74 eV) semiconductor with a suitable band potential for water splitting [55]. The bottom of the conduction band is located at a potential more negative than the reduction potential of H^+^ to H_2_. Au nanocrystals have also been shown to possess the activity for catalytic reaction [41]. On the one hand, the surface plasmon of Au could effectively harvest the light energy and decay to energetic carriers. On the other hand, the plasmon-enhanced local field enhances the light absorption of adjacent CdSe [56]. These effects would improve the generation of photoexcited carriers for the photocatalytic reactions. Then the photoexcited electrons/holes should be separated and migrated to the surface without recombination. Since holes and electrons respectively gain energy by moving up and down, the photoexcited electrons can be transferred from the conduction band (CB) of the CdSe to the Fermi level of Au. The charge transfer across the interface between the CdSe and Au play a critical role for achieving this goal and accelerating the yield of H_2_ generation [41–44]. The conditions of interface and contact between the two components determine the charge transfer performance and thus the photocatalytic properties of hybrids. Compared with the multi-head structure, the H_2_ production efficiency of the single-head structure (heterodimers and Janus nanospheres) is higher. When more CdSe heads are grown onto Au, more Au surfaces acting as the reaction site would be blocked from the reaction solution. Compared with the other three heterostructures, the Au/CdSe Janus nanospheres exhibit a flat interface with high-degree crystallization and low interfacial strain, which could improve the interfacial charge transfer efficiency and suppress the carrier scattering loss. The size of plasmonic nanoparticles, the morphology of the hybrids, the dimension of semiconductor component, and the position of catalytic active sites are all critical for the photocatalytic activity [41, 44]. The optimal dimension of Janus Au/CdSe for the photocatalytic application need to be further investigated.

## Conclusion

In summary, we presented a precise synthesis of water-dispersed Au/CdSe Janus nanospheres with controlled interfacial condition and quality. Four types of Au/CdSe hybrids of Janus nanospheres, heterodimers, symmetric double-headed nanoparticles, and multi-headed nanoparticles could be produced by manipulating the pH value. The evaluation of photocatalytic hydrogen generation showed that the Au/CdSe Janus nanospheres exhibit at least 3.9 times higher H_2_ evolution rate than other Au/CdSe counterparts. The improved photocatalytic performance is owing to the flat and high-quality interface between Au and CdSe, which promotes the charge transfer across the interface and accelerates the interfacial charge separation.

## Supplementary information

**Additional file 1: Figure S1**. The HRTEM images of different types of Au/CdSe hybrid nanoparticles such as (a) Janus nanospheres, (b) heterodimers, (c) symmetric double-headed nanoparticles.

## Data Availability

All data generated or analyzed during this study are included in this article and its supplementary information file.

## Abbreviations

XRD: X-ray powder diffraction
EDX: Energy-dispersive X-ray spectroscopy
TEM: Transmission electron microscopy
HRTEM: High-resolution transmission electron microscope
