# Supplementary material for: Synthesis of Au/CdSe Janus Nanoparticles with Efficient Charge Transfer for Improving Photocatalytic Hydrogen Generation
Source: Nanoscale Res Lett. 2019 Nov 27;14:349. doi: 10.1186/s11671-019-3185-6 (PMC6881496; doi:10.1186/s11671-019-3185-6)
Supplement: Supplementary file 1 — Additional file 1: Figure S1. The HRTEM images of different types of Au/CdSe hybrid nanoparticles such as (a) Janus nanospheres, (b) heterodimers, (c) symmetric double-headed nanoparticles. [file 11671_2019_3185_MOESM1_ESM.doc]

**Supporting Information**

Synthesis of Au/CdSe Janus nanoparticles with efficient charge transfer for improving photocatalytic hydrogen generation

Xiao-Dan Liu^1^, Kai Chen^2^, Song Ma^1^, Zhong-Hua Hao^1^, Shan Liang^3*^, Li Zhou^1*^, and Qu-Quan Wang^1,2*^

^1^Key Laboratory of Artificial Micro- and Nano-structures of the Ministry of Education, School of Physics and Technology, Wuhan University, Wuhan 430072, People’s Republic of China

^2^Department of The Institute for Advanced Studies, Wuhan University, Wuhan 430072, People’s Republic of China

^3^Department of Physics, Hunan Normal University, Changsha, 410081, People’s Republic of China

Email: [xdliu_opt@126.com](mailto:xdliu_opt@126.com) (X. D. Liu); [kchen_opt@126.com](mailto:kchen_opt@126.com) (K. Chen); [sma_opt@126.com](mailto:sma_opt@126.com) (S. Ma); zhhao@whu.edu.cn (Z. H. Hao); [liangshan@hunnu.edu.cn](mailto:liangshan@hunnu.edu.cn) (S. Liang); zhouli@whu.edu.cn (L. Zhou); qqwang@whu.edu.cn (Q.-Q. Wang).

^*^Correspondence: [liangshan@hunnu.edu.cn](mailto:liangshan@hunnu.edu.cn); [zhouli@whu.edu.cn](mailto:zhouli@whu.edu.cn); qqwang@whu.edu.cn


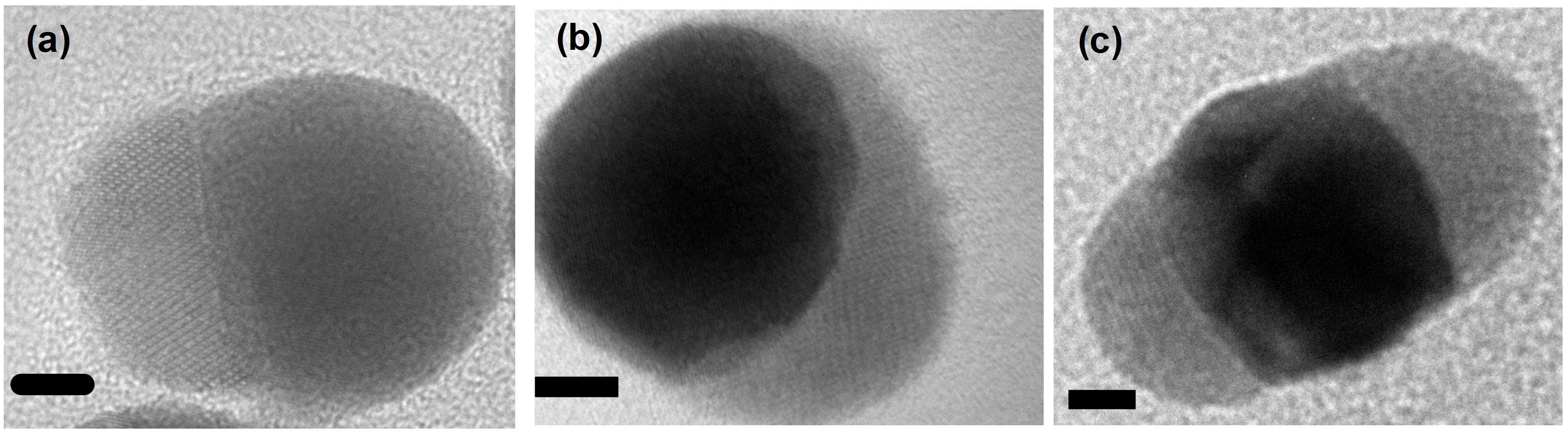


**Figure S1. The HRTEM images of different types of Au/CdSe hybrid nanoparticles such as (a) Janus nanospheres, (b) heterodimers, (c) symmetric double-headed nanoparticles. The scale bars are 5 nm.**
